# Protective role of melatonin in early‐stage and end‐stage liver cirrhosis

**DOI:** 10.1111/jcmm.14634

**Published:** 2019-09-02

**Authors:** Chenxia Hu, Lingfei Zhao, Jingjing Tao, Lanjuan Li

**Affiliations:** ^1^ Collaborative Innovation Center for Diagnosis and Treatment of Infectious Diseases, State Key Laboratory for Diagnosis and Treatment of Infectious Diseases, School of Medicine, First Affiliated Hospital Zhejiang University Hangzhou Zhejiang China; ^2^ Kidney Disease Center, College of Medicine, First Affiliated Hospital Zhejiang University Hangzhou Zhejiang China; ^3^ Key Laboratory of Kidney Disease Prevention and Control Technology Hangzhou Zhejiang China; ^4^ Institute of Nephrology Zhejiang University Hangzhou Zhejiang China

**Keywords:** hepatic stellate cells, liver cirrhosis, melatonin, oxidative stress, regression

## Abstract

The liver is composed of hepatocytes, cholangiocytes, Kupffer cells, sinusoidal endothelial cells, hepatic stellate cells (HSCs) and dendritic cells; all these functional and interstitial cells contribute to the synthesis and secretion functions of liver tissue. However, various hepatotoxic factors including infection, chemicals, high‐fat diet consumption, surgical procedures and genetic mutations, as well as biliary tract diseases such as sclerosing cholangitis and bile duct ligation, ultimately progress into liver cirrhosis after activation of fibrogenesis. Melatonin (MT), a special hormone isolated from the pineal gland, participates in regulating multiple physiological functions including sleep promotion, circadian rhythms and neuroendocrine processes. Current evidence shows that MT protects against liver injury by inhibiting oxidation, inflammation, HSC proliferation and hepatocyte apoptosis, thereby inhibiting the progression of liver cirrhosis. In this review, we summarize the circadian rhythm of liver cirrhosis and its potential mechanisms as well as the therapeutic effects of MT on liver cirrhosis and earlier‐stage liver diseases including liver steatosis, nonalcoholic fatty liver disease and liver fibrosis. Given that MT is an antioxidative and anti‐inflammatory agent that is effective in eliminating liver injury, it is a potential agent with which to reverse liver cirrhosis in its early stage.

## 1 BACKGROUND

The liver is the largest organ with synthesis and secretion functions and is composed of hepatocytes, cholangiocytes, Kupffer cells (KCs), sinusoidal endothelial cells, hepatic stellate cells (HSCs) and dendritic cells, among others.^1^ Liver cirrhosis is the end‐stage disease of liver disease induced by various hepatotoxic factors including infection, chemicals, high‐fat diet (HFD) consumption, surgical procedures and genetic mutation, as well as biliary tract diseases such as sclerosing cholangitis and bile duct ligation (BDL).^2^ Chronic liver injury impairs epithelial cells and triggers a fibrogenic response to recruit inflammatory cells (eg, macrophages and T cells), consequently inducing the activation and proliferation of extracellular matrix (ECM)‐producing cells including fibroblasts and HSCs in liver tissue. The subsequent collagen deposition process gives rise to uncontrolled wound‐healing pathophysiology and irreversible formation of intrahepatic scar tissue.^3^, ^4^ After liver fibrosis progresses into the end stage, multiple complications including acute or chronic liver failure, portal hypertension and hepatocarcinoma will follow.

In liver tissue, superoxide anions, hydrogen peroxide and hydroxyl radicals can be converted into stable reactive oxygen species (ROS) with strong toxicity, such as nitric oxide and peroxynitrites.^5^ Thus, therapies targeting ROS inhibition are in great demand for inhibiting injury caused by oxidative stress and improving the prognosis of liver cirrhosis. Melatonin (MT), also known as *N*‐acetyl‐5‐methoxytryptamine, is isolated from the pineal gland and participates in regulating multiple physiological functions including sleep promotion, circadian rhythms and neuroendocrine processes.^6^, ^7^, ^8^ In the pineal gland, tryptophan is hydroxylated by tryptophan‐5‐hydroxylase to generate 5‐hydroxytryptophan, which is then decarboxylated into 5‐hydroxytryptamine (serotonin) by l‐aromatic amino acid decarboxylase. After that, the serotonin is acetylated to generate *N*‐acetylserotonin, which is finally converted to MT.^9^ The hydroxylated MT generated by hepatic cytochrome P_450_ mono‐oxygenases is conjugated with sulphate to generate active 6‐sulfatoxymelatonin.^10^ MT not only exerts a strong antioxidant effect to protect cells and tissues from radical damage^11^ but also inhibits proinflammatory cytokines including tumour necrosis factor (TNF)‐α, interleukin (IL)‐1β and IL‐6 b decreasing NF‐κB during the development of hepatic fibrosis.^12^, ^13^ MT dramatically inhibits the activation of leucocyte, macrophage, mononuclear cells, mast cell and neutrophil infiltration in animal models of liver fibrosis.^14^ Most importantly, MT contributes to a reduction in the amount of ECM deposition and significantly reduces histopathological changes in liver tissue.^15^

**Figure 1:**
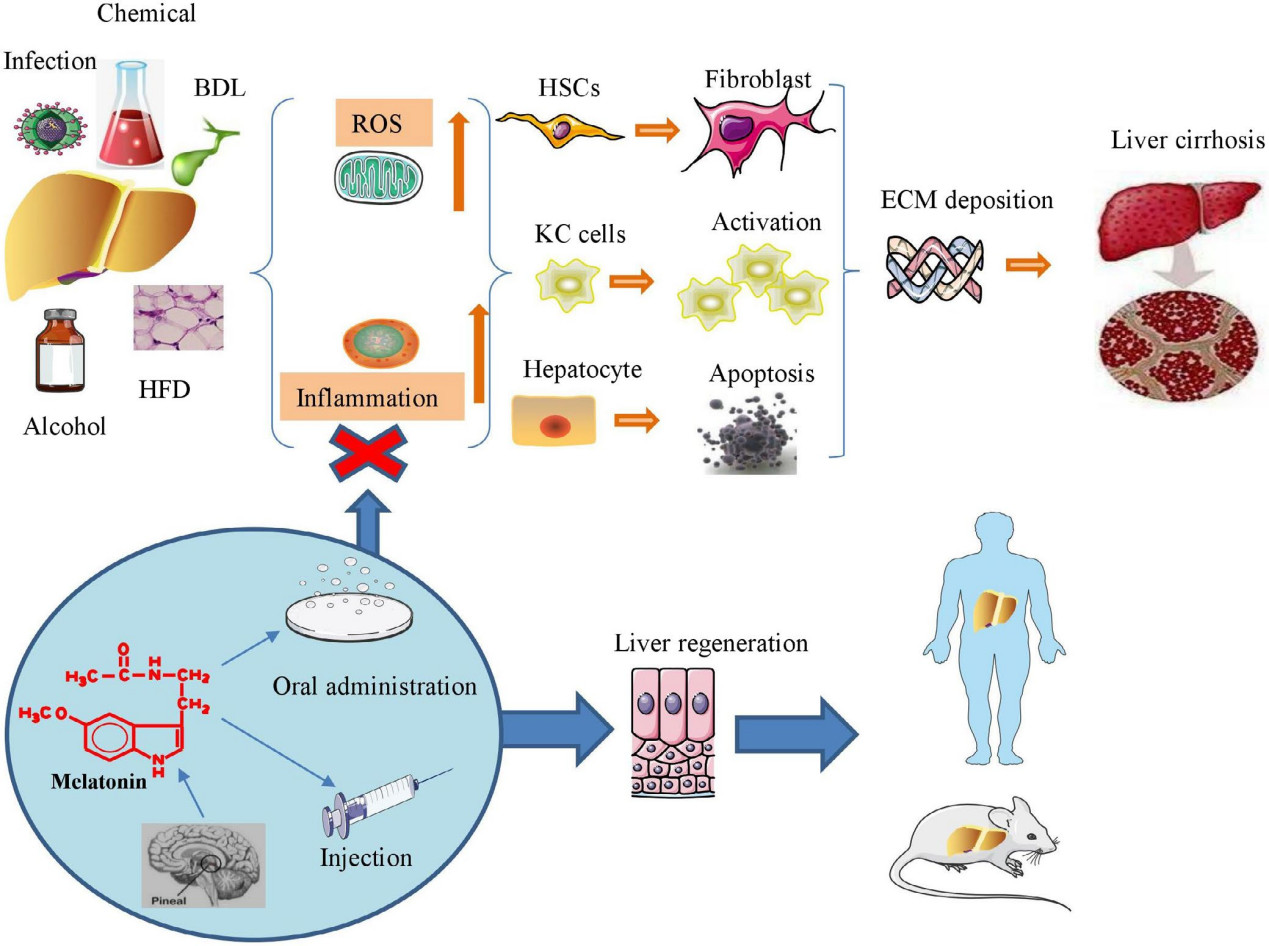
Melatonin reduces oxidative stress and inflammation to eliminate collagen deposition and prevent fibrosis progression

This review summarizes the circadian rhythm of liver cirrhosis and its potential mechanisms, as well as the therapeutic effects of MT on liver cirrhosis and earlier‐stage liver diseases including liver steatosis, nonalcoholic fatty liver disease (NAFLD) and liver fibrosis (Figure 1). We describe how MT protects against liver injury against liver injury by inhibiting oxidation, inflammation, HSC proliferation and hepatocyte apoptosis, thereby inhibiting progression of liver cirrhosis. When the optimal dose, time‐point, route and duration of administration are determined for MT to target oxidative liver damage in animal models and human beings, it may become a standard agent for liver disease treatment.

## 2 THE POTENTIAL MECHANISMS OF MT TARGETING LIVER CIRRHOSIS

**Figure 2:**
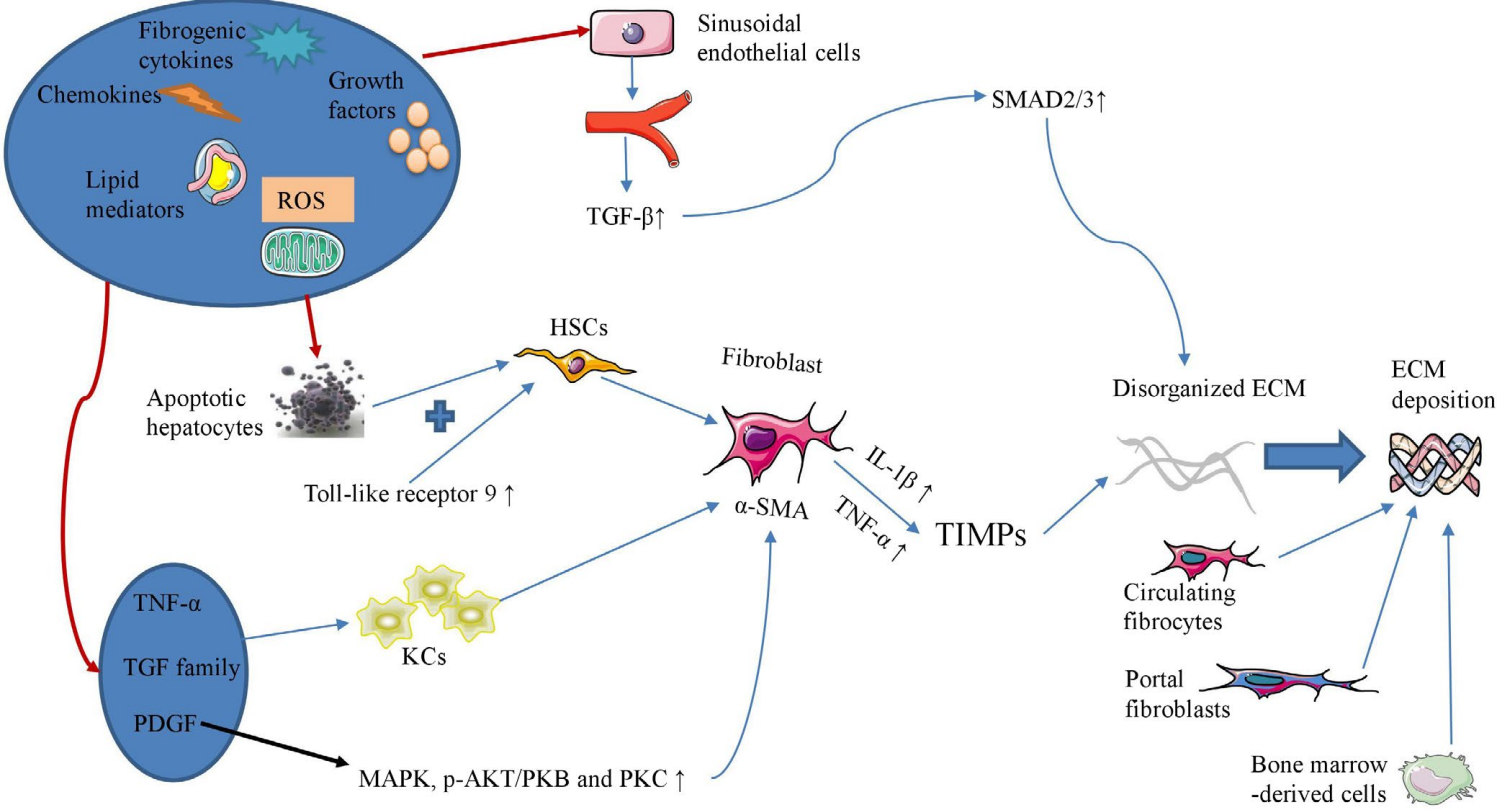
Several pathogenetic factors and pathways participate in the initiation and progression of liver cirrhosis

Multiple fibrogenic cytokines, chemokines, growth factors and lipid mediators as well as ROS production promote the transdifferentiation of quiescent HSCs into a myofibroblastic phenotype, and the interaction between apoptotic hepatocytes and Toll‐like receptor 9 also induces HSCs to transdifferentiate into myofibroblasts.^16^, ^17^ On the other hand, the capillarization of sinusoidal endothelial cells up‐regulates transforming growth factor (TGF)‐β levels, transdifferentiates HSCs into activated myofibroblasts, and triggers hepatocyte apoptosis.^18^, ^19^ TGF‐β interacts with the type II and type I receptors to activate small mothers against decapentaplegic (SMAD)2/3 and initiate collagen deposition in liver tissue.^20^ In addition, activated HSCs produce tissue inhibitors of metalloproteinases (TIMPs) and induce ECM deposition in liver tissue via up‐regulation of IL‐1β and TNF‐α.^21^ Then, the harsh microenvironment up‐regulates the expression of inflammatory cytokines including TNF‐α, the TGF family and platelet‐derived growth factor (PDGF), activating KCs and aggravating the progression of liver fibrosis.^20^ The PDGF‐β signalling pathway subsequently activates other signalling pathways including the MAPK, p‐AKT/PKB and PKC pathways to enhance the proliferation and fibrogenesis of HSCs.^22^ In addition to HSCs and KCs, circulating fibrocytes, portal fibroblasts and bone marrow‐derived cells also take part in ECM deposition during liver fibrosis.^23^, ^24^ Although excessive ROS has been demonstrated to up‐regulate the cell death rate of HSCs, nontoxic ROS is able to promote the activation, proliferation and collagen production of HSCs.^25^ After the balance of matrix metalloproteinases (MMPs) and TIMPs is damaged, the deposition of scar tissue will result in irreversible liver fibrosis even if the stimulating factors are withdrawn.^26^ Therapeutic approaches targeting elimination of liver fibrosis should interrupt each step, such as inflammation, hepatocyte apoptosis, cholangiocyte proliferation, myofibroblast activation and ECM deposition.^27^ As MT exhibits strong antioxidant activity and inhibits inflammation, HSC proliferation and hepatocyte apoptosis, interventions targeting the pathogenetic factors or pathways in this manner will help to block the initiation and progression of liver cirrhosis (Figure 2).

## 3 CIRCADIAN RHYTHM IN PATIENTS WITH LIVER CIRRHOSIS AND HEPATIC ENCEPHALOPATHY

Melatonin is generally regulated by light and darkness in both diurnal and nocturnal animals, as light eliminates MT synthesis, whereas darkness, peaking in the middle of the night, permits MT synthesis. The central MT synthesis pattern has been shown to be disturbed in patients with hepatic cirrhosis and is correlated with the severity of liver injury. Patients with liver cirrhosis had markedly elevated MT levels during daytime hours, and both the onset time of the MT increase and the time of the peak MT concentration were consistently and significantly delayed in these patients.^28^ The elevated daytime MT levels in patients with liver cirrhosis are probably related to a decreased metabolic clearance rate, decreased liver blood flow, lowered activity of 6‐β‐hydroxylase and competition with bilirubin in the intrahepatic transport system.^29^ The disturbed circadian 6‐sulfatoxymelatonin rhythm impairs the quality of nighttime sleep and increases daytime sleepiness in patients with liver cirrhosis, and bright light therapy has been demonstrated to exert no beneficial effects on them, perhaps due to the severity of central circadian disruption at baseline.^30^ However, there is a controversy about the relationship between circadian abnormalities and impaired sleep quality. Montagnese et al found that although patients with liver cirrhosis demonstrated delayed peak serum MT, the urinary 6‐sulfatoxymelatonin levels of patients and healthy volunteers did not differ. They concluded that there was no association between circadian abnormalities and impaired sleep quality.^31^

Liver cirrhosis induces a type of brain dysfunction, namely, hepatic encephalopathy (HE), which includes multiple symptoms ranging from subclinical alterations to coma with or without neurological or psychiatric abnormalities.^32^ Early in 1954, Sherlock found that there existed a so‐called sleep‐wake inversion, which combined restless nights and excessive daytime sleepiness in patients with HE induced by liver cirrhosis.^33^ A considerable proportion of patients with cirrhosis have the complication of HE with seriously impaired hepatic MT metabolism, but patients with subclinical HE showed impairment of life activities without clinical neurologic abnormalities. However, the understanding of their pathophysiology remains limited, and their treatment is problematic. Abnormal pituitary hormone and MT circadian patterns are present in liver cirrhosis before development into HE, and MT is the only hormone associated with the severity of liver insufficiency and serves as an early indicator of impending HE.^34^ After fasting treatment, MT was detected in ascitic fluid in multiple patients with HE, and the high levels of MT in these patients may account for some of the clinical manifestations of HE including daytime sleepiness and fatigue.^35^ Chojnacki et al enrolled 75 patients with alcohol‐induced liver cirrhosis and 25 healthy control individuals; they found that the levels of serotonin, urinary 5‐hydroxyindoleacetic acid and 6‐sulfatoxymelatonin were lowest while the level of MT was highest in patients with Child‐Pugh C grade, and the disturbance of serotonin and MT homeostasis in patients with liver cirrhosis may be associated with advanced HE.^36^ However, although ammonia level is highest in patients with grade 3 HE, there is no correlation between MT and ammonia levels in these patients.^37^

## 4 MT APPLICATION IN CLINICAL TRIALS

The fasting and postprandial plasma MT levels and portal hypertension rose significantly after treatment with MT or tryptophan, particularly in liver cirrhosis patients, which is attributable to portal systemic shunting and decreased liver degradation.^38^

Nonalcoholic steatohepatitis (NASH) patients underwent treatment with Essentiale Forte and tryptophan or MT for 4 weeks demonstrated reduced expression levels of γ‐glutamyl transpeptidase (GGTP), triglycerides and proinflammatory cytokines including IL‐1, IL‐6 and TNF‐α, while these patients showed no significant alteration in alanine transaminase (ALT) level.^39^ In addition, NAFLD patients underwent treatment with Essentiale Forte and tryptophan or MT for 14 months demonstrated reduced expression levels of GGTP, triglycerides, low‐density lipoprotein cholesterol and proinflammatory cytokines including IL‐1, IL‐6 and TNF‐α, while these patients demonstrated no significant difference in ALT level and other biochemical parameters.^40^ A 12‐week course of MT can not only reduce levels of liver enzymes during the treatment period but also maintain the alterations after discontinuation in patients with NASH.^41^ Pakravan et al enrolled 100 patients with NAFLD for 3 months of MT treatment and found that the levels of diastolic blood pressure, aspartate aminotransferase (AST) and high‐sensitivity C‐reactive protein were significantly lower and the liver grades better in patients with NAFLD who received MT than in those received placebo.^42^

## 5 MT EFFECTIVELY REDUCES LIVER INJURY IN ANIMAL MODELS OF LIVER CIRRHOSIS

### 5.1 Chemically induced liver fibrosis

Chemicals including carbon tetrachloride (CCl_4_), thioacetamide (TAA) and dimethylnitrosamine (DMN) are commonly used to generate animal models of liver cirrhosis to investigate the effects of MT on reversing liver fibrosis (Table 1).

**Table 1:** The potential mechanisms through which MT may attenuate chemically induced liver cirrhosis

Chemical type	Animal	MT dose	Time	Effect	Mechanism	Ref.
CCl_4_	Rat	5, 10 and 20 mg/kg/d	6 wk	Decreases serum transaminase activity; reduces liver fibrosis scores	NF‐κB in liver tissue and proinflammatory cytokines such as TNF‐α and IL‐1β in KC cells	16
CCl_4_	Rat	25 mg/kg/d	1 mo	Reduces injury	Oxidative stress↓; Bax↓	46
CCl_4_	Mouse	5 or 10 mg/kg/d	2 or 4 wk	Prevents all pathological changes; alleviate the progression of liver fibrosis	HSC activity↓; differentiation of HSCs into myofibroblasts↓	47
CCl_4_	Mouse	5 or 10 mg/kg/d	2 or 4 wk	Abrogates the activation of HSCs, maintains normal histopathology; decreases levels of serum transaminases	Profibrogenic genes↓; MMP‐9↓; Nrf2↑	48
CCl_4_	Mouse	5 or 10 mg/kg/d	2 or 4 wk	Down‐regulates the levels of TGF‐β and collagen Ι	SphK1/S1P axis↓	49
CCl_4_	Rat	20 mg/kg/d	6 wk	Decreases the deterioration of liver cirrhosis	NF‐κB/p65↓; iNOS↓; inflammatory infiltrate↓; angiogenesis↓	50
CCl_4_	Rat	2.5, 5, and 10 mg/kg/d	8 wk	Reduces levels of hepatic hydroxyproline; reduces hepatocyte apoptosis; decreases HSC activation	Necroptosis‐associated inflammatory signalling↓; damage‐associated molecular patterns↓; (Toll‐like receptor 4 expression, p38, c‐Jun N‐terminal kinase phosphorylation, and NF‐κB translocation)↓	51
CCl_4_	Mouse	5 or 10 mg/kg/d	2 or 4 wk	Abrogates HSC activation	Autophagic response↓; ER↓; phospho‐IRE1↓; ATF6↓; phospho‐PERK↓	52
CCl_4_	Rat	2.5, 5, and 10 mg/kg/d	8 wk		Mitochondrial dysfunction↓; mitochondrial swelling↓; glutamate dehydrogenase release↓	53
CCl_4_	Rat	20 mg/kg/d	1 mo	Decreases the levels of lipid deposition, ALT and hydroxyproline; increases the level of albumin	Oxidative stress↓; matrix balance↑	54
TAA	Rat	5 mg/kg/d	Approximately 9 wk	Inhibits excessive oxidative stress	Thioredoxin‐1↑; autotaxin↓	55
TAA	Rat	10 mg/kg/d	4 wk	Decreases the levels of liver enzymes and proinflammatory cytokines	PON‐1↑; GSH↑; GSSG↓	56
TAA	Rat	1 mg/kg/d	1 or 3 mo	Eliminates HSC activation and extensive tissue damage	Oxidative stress↓; α‐SMA↓	57
DMN	Rat	100 mg/kg/d	2 wk	Suppresses hepatic fibrotic changes, but exerts no effects on changes in biochemical parameters when administered alone	Hydroxyproline↑; MDA↑; GSH↓; SOD↓	58

Abbreviations: α‐SMA, α‐smooth muscle actin; ALT, alanine transaminase; CCl_4_, carbon tetrachloride; DMN, dimethylnitrosamine; ER, endoplasmic reticulum; GSH, glutathione; HSC, hepatic stellate cell; iNOS, inducible nitric oxide synthase; KC, Kupffer cell; IL, interleukin; MMP, matrix metalloproteinase; MDA, malondialdehyde; MT, melatonin; SOD, superoxide dismutase; TAA, thioacetamide; TGF, transforming growth factor; TNF, tumour necrosis factor.

A majority of such studies have focused on the effects of MT on reversing CCl_4_‐induced liver cirrhosis in animal models and further clarified the underlying mechanisms as follows. MT was demonstrated to significantly attenuate oxidative stress‐induced injury and the expression of Bax and other apoptotic proteins in CCl_4_‐induced liver fibrosis.^43^ Furthermore, Hong et al^15^ demonstrated that a high dose of MT significantly reduced the serum levels of laminin, hyaluronic acid and hydroxyproline, thus attenuating liver fibrogenesis in a dose‐dependent manner. In addition to down‐regulating α‐smooth muscle actin (α‐SMA) and up‐regulating peroxisome proliferator‐activated receptor alpha (PPARα), MT up‐regulated expression of brain and muscle Arnt‐like protein 1 (BMAL1), circadian locomotor output cycles kaput (CLOCK), period 2 (PER2), cryptochrome 1 (CRY1) and RAR‐related orphan receptor‐α (RORα) to maintain the circadian clock machinery in CCl_4_‐treated mice.^44^ MT treatment significantly abolished the activation of HSCs and increased the expression of nuclear factor erythroid‐2‐related factor 2 (Nrf2) while inhibiting the expression of profibrogenic genes such as MMP‐9, collagens I and III, TGF‐β, PDGF, connective tissue growth factor, amphiregulin and phospho‐SMAD3 in mice with CCl_4_‐induced liver fibrosis.^45^ In addition, the levels of NF‐κB in liver tissue and the levels of proinflammatory cytokines such as TNF‐α and IL‐1β released from KCs were down‐regulated in rats with CCl_4_‐induced fibrosis.^13^ An important pathway, the sphingosine kinase 1/sphingosine 1‐phosphate (SphK1/S1P) axis, can also be inhibited by MT during the development of liver fibrogenesis.^46^ After a 6‐week treatment, MT not only significantly reduced the inflammatory process as shown by decreased levels of NF‐κB/p65, inducible nitric oxide synthase and inflammatory infiltrate but also significantly decreased angiogenesis as demonstrated by reduced expression of TGF‐β1, α‐SMA and vascular endothelial growth factor, subsequently slowing the deterioration of liver cirrhosis.^47^ MT also attenuated CCl_4_‐induced liver fibrosis not only by inhibiting necroptosis‐associated inflammatory signalling including the necrosome complex, RIP1, RIP3 and the downstream effector (MLKL) but also by decreasing the levels of damage‐associated molecular patterns including high‐mobility group box 1, IL‐1α and IL‐33. Other signalling events including Toll‐like receptor 4 binding, p38 activity, c‐Jun N‐terminal kinase phosphorylation and NF‐κB translocation were also down‐regulated to reduce hepatocyte apoptosis and decreased HSC activation in rats with liver fibrosis after MT administration.^48^ Autophagy is a process of programmed cell death and can be activated by CCl_4_ administration in mice, while MT treatment significantly inhibited autophagy and reduced the endoplasmic reticulum (ER) stress accompanied with down‐regulation of phospho‐IRE1, ATF6 and phospho‐PERK protein.^49^ Chronic CCl_4_ exposure led to impaired mitophagy (autophagy in mitochondria) and disturbed mitochondrial biogenesis and mitochondrial dysfunction, while MT attenuated hallmarks of mitochondrial dysfunction as demonstrated by up‐regulation of mitochondrial DNA, PTEN‐induced putative kinase 1 (PINK1), Parkin, peroxisome proliferator‐activated receptor‐γ coactivator 1α (PGC‐1α), nuclear respiratory factor 1 (NRF1) and mitochondrial transcription factor A (TFAM).^50^ In an intriguing study, Mortezaee et al injected MT after the last dose of CCl_4_ in rats for 1 month and set this group as the post‐treatment group. They suggested that MT post‐treatment was more powerful than cotreatment in reducing liver fibrosis via reduction of oxidative stress and maintenance of matrix balance, including up‐regulation of MMP‐13 and Bcl2.^51^

Chronic administration of TAA significantly decreased superoxide dismutase (SOD) and glutathione (GSH) activity but increased the hepatic content of malondialdehyde (MDA), which is the end product of lipid peroxidation, thus causing excessive oxidative stress in liver tissue. However, MT exhibits anti‐inflammatory, antioxidant and fibrosuppressive activity against hepatic fibrogenesis via induction of thioredoxin‐1 and elimination of autotaxin.^52^ MT also serves as a mediator of oxidative stress and protects the liver from TAA‐induced injury by increasing the levels of paraoxonase 1 (PON‐1) and GSH and inhibiting the activity of oxidized glutathione (GSSG), consequently decreasing the levels of liver enzymes and proinflammatory cytokines.^53^ MT eliminated HSC activation and abrogated TAA‐induced oxidative stress, along with the extensive tissue damage that accompanies it, in rats with hepatic fibrosis.^54^ On the other hand, although MT exerts no additional effects on normal rats, it effectively attenuated the hepatic fibrotic changes in a DMN‐induced liver fibrosis model by down‐regulating hydroxyproline and MDA and up‐regulating GSH and SOD.^55^

### 5.2 Liver fibrosis induced by biliary tract disease

The pathogenesis of primary biliary cirrhosis, primary sclerosing cholangitis (PSC) and autoimmune hepatitis is different from that of chronic liver fibrosis induced by toxic factors, as portal fibroblasts are found around bile ducts, but not as a result of HSC activation in response to profibrogenic or mitogenic stimuli.^56^

Manifestations of primary biliary cirrhosis include increased skin melanin pigmentation, elevated cholesterol and alkaline phosphatase levels, defective T lymphocytes and hyperactive B lymphocytes and hepatic fibrosis.^57^, ^58^, ^59^, ^60^ After exposure to darkness or administration of MT, multidrug resistance gene 2‐knockout (Mdr2^−/−^) mice with primary sclerosing cholangitis showed elevated serum MT level and decreased biliary mass, along with reduction of liver fibrosis and angiogenesis.^61^ In addition, MT decreased the mean severity scores of liver parenchymal necrosis, portal fibrosis, biliary duct proliferation and cholangitis in rats with formalin‐induced sclerosing cholangitis.^62^

Cholangiocyte proliferation is initiated after BDL at the edge of the portal tract and caused the generation of proliferating bile ductules with portal inflammation and fibrosis; the pathological changes in the liver after BDL are more severe at 4 weeks than at 2 weeks.^63^ Fortunately, MT exerts protective effects against BDL‐induced injury and inhibits the progression of liver fibrosis (Table 2). MT significantly decreased the expression of clock genes (PER1, BMAL1, CRY1 and CLOCK) as well as cAMP levels and PKA phosphorylation, consequently reducing serum bilirubin and transaminase levels, the percentage of PCNA‐positive cholangiocytes and biliary hyperplasia in the cholangiocytes of BDL rats.^64^ After administration in vivo, MT was effective in reducing the hepatosomatic and splenosomatic indices, restoring normal levels of lipid peroxidation and antioxidant enzyme expression and inhibiting inflammation, thus decreasing liver injury and liver fibrosis in BDL‐induced secondary biliary cirrhosis.^65^ On the other hand, MT is able to suppress the release of hypothalamic gonadotropin‐releasing hormone (GnRH), a hormone that promotes cholangiocyte proliferation.^65^ McMillin et al also concluded that MT alleviated liver injury in cholestatic rats via GnRH receptor 1‐dependent paracrine signalling and demonstrated that supernatants from cholangiocytes isolated from BDL rats infused with MT suppressed the activation of HSCs induced by BDL cholangiocyte supernatant.^66^ BDL not only increased collagen deposition but also improved MDA expression and luminol and lucigenin signal while decreasing GSH levels, whereas MT serves as a powerful physiological scavenger of hydroxyl radicals to reverse the activation of HSCs.^67^ BDL increased the prevalence of kidney and brain injury in animal models. BDL increased the activity of hepatic dimethylarginine dimethylaminohydrolase (DDAH), which serves as an asymmetric dimethylarginine (ADMA)‐metabolizing enzyme in both the liver and kidney of rats, while MT therapy effectively decreased mortality and prevented kidney injury characterized as measured by decreased tubulointerstitial injury scores and plasma creatinine and symmetric dimethylarginine levels in BDL rats.^68^ Intriguingly, MT maintained brain‐derived neurotrophic factor in the dorsal hippocampus of young BDL rats at a level comparable to that of sham controls, consequently preventing spatial deficits and decreasing ADMA levels in the plasma, prefrontal cortex and dorsal hippocampus of these cholestatic rats.^69^ However, the dose of MT may influence the outcome of clinical symptoms because the antioxidative stress capacity varies according to the MT dose. Although low‐dose and high‐dose MT treatment both significantly improved the plasma MDA levels, liver MDA levels and liver GSH/GSSG ratios in BDL rats, only a high dose of MT (1000 µg/kg/d) was able to improve the spatial performance of rats with BDL‐induced liver fibrosis.^70^

**Table 2:** The potential mechanisms through which MT may attenuate BDL‐induced liver cirrhosis

Animal	Dose	Time	Route	Effect	Mechanism	Ref.
Rat	2 mg/g/d	1 wk	Per os	Decreases serum bilirubin and transaminase levels; decreases the percentage of PCNA‐positive cholangiocytes; inhibits biliary hyperplasia	cAMP↓; clock genes↓; PKA phosphorylation↓; basal bile and bicarbonate secretion↓	67
Rat	20 mg/kg/d	2 wk	Intraperitoneal	Reduces the hepatosomatic and splenosomatic indices; decreases cholangiocyte proliferation; decreases liver injury and liver fibrosis	Lipid peroxidation↑; antioxidant enzymes↑; inflammation↓; GnRH↓; iNOS↓; TNF‐α↓	68
Rat	1 mg/kg/d	7 d	Intracerebroventricular (ICV) cannulas	Alleviates liver injury in cholestatic rats; reduces cholangiocyte proliferation; alleviates fibrosis	Hypothalamic and cholangiocyte GnRH↓	69
Rat	100 mg/kg/d	1 mo	Intraperitoneal	Reverses HSC activation	MDA↓; luminal signal↓; lucigenin signal↓; GSH↑	70
Rat	1 mg/kg/d	14 d	Intraperitoneal	Decreases mortality; prevents kidney injury	Hepatic DDAH2 expression↑, DDAH activity↑; ADMA contents in both the liver and the kidney↓	71
Rat	5 mg/d	4 wk	A slow‐release melatonin pellet implanted in the peritoneum	Prevents spatial deficits; decreases ADMA levels in the plasma, prefrontal cortex and dorsal hippocampus	Maintains brain‐derived neurotrophic factor in the dorsal hippocampus	72
Rat	500/1000 µg/kg/d	2 wk	Intraperitoneal	Improve spatial performance of rats with BDL‐induced liver fibrosis	Plasma MDA↑; liver MDA↑; liver GSH/GSSG ratios↑	73

Abbreviations: BDL, bile duct ligation; GnRH, gonadotropin‐releasing hormone; GSH, glutathione; HSC, hepatic stellate cell; iNOS, inducible nitric oxide synthase; MDA, malondialdehyde; MT, melatonin; TNF, tumour necrosis factor.

### 5.3 NAFLD and NASH

Nonalcoholic fatty liver disease, a state ranging from simple steatosis to steatohepatitis, advanced fibrosis and cirrhosis, is attributed to specific dietary habits and lifestyles and results in liver dysfunction and end‐stage liver cirrhosis. In particular, NASH, which is currently recognized as a serious condition, may also progress to liver cirrhosis because of pathophysiological mechanisms including oxidative stress, lipid peroxidation and excessive hepatocyte apoptosis.^12^ Lipid infiltration results in mitochondrial fission and mitochondrial fragments in hepatocytes by disrupting the interaction of SIRT1 and Mitofusin 2, consequently up‐regulating glycolytic flux, mitochondrial permeability transition pore (mPTP) opening, ROS production, cell cycle arrest and apoptosis of hepatocytes.^71^ Primary hepatocytes isolated from mice with HFD‐induced NAFLD showed up‐regulated NR4A1 levels and activation of DNA‐PKcs and p53, which up‐regulated Drp1‐mediated mitochondrial fission and mitophagy arrest. Thus, the cultured hepatocytes demonstrated mitochondrial dysfunction including extensive mPTP opening, decreased mitochondrial potential, oxidative stress, calcium overload, mitochondrial respiratory collapse and ATP shortage.^72^

An HFD significantly induced oxidative stress and up‐regulated the levels of serum ALT, serum AST, total liver cholesterol and liver triglycerides in NAFLD rats.^73^ Application of MT significantly reduces the pathogenetic changes in animal models with NAFLD and NASH according to current evidence (Table 3). Although application with MT did not alter the levels of lipids among HFD rats, MT is demonstrated to effectively reduce liver weight, the ratio of liver weight to bodyweight, portal vein pressure, the expression of HFD‐induced plasma protein related to liver steatosis, the necrosis rate of liver cells and the extent of liver damage in animal models.^74^, ^75^ Others debated whether MT administration significantly reduced lipid accumulation in vivo to reduce the progression of liver fibrosis via different pathways. MT abolishes lipotoxicity‐mediated HSC activation and prevents HFD‐induced fibrosis through up‐regulation of the mitochondrial respiratory chain and the tricarboxylic acid cycle (TCA) cycle.^71^ MT promotes the loss of lipid droplets by directly suppressing HSC activation in vitro and enhancing signalling through RORα, which serves as the nuclear MT sensor in quiescent and activated HSCs, in a dose‐dependent manner.^76^ MT can regulate lipid metabolism, increase insulin sensitivity, regulate glucose metabolism and reduce ALT, low‐density cholesterol bodyweight and liver weights in HFD‐fed mice. The protection was mediated by down‐regulation of TNF‐α, IL‐1β, and IL‐6 and decreased phosphorylation of P38 and JNK1/2^77^; moreover, MT significantly ameliorated lipid deposition by down‐regulating ER stress and up‐regulating AKT phosphorylation and fetuin‐A expression.^78^ MT reversed the pathological progress of NAFLD by reducing hyperglycaemia and metabolic dysfunction and restoring mitochondrial function as well as the cellular longevity of hepatocytes in vivo.^79^ Pioglitazone (an insulin sensitizer), pentoxifylline (a TNF‐α inhibitor) and MT (an antioxidant) alone or in combination reduced severe hepatic steatosis, inflammation and fibrosis in rats with NAFLD. Pentoxifylline was demonstrated to decrease serum TNF‐α levels, while pioglitazone and MT significantly reduced serum total cholesterol and triglycerides by down‐regulating MDA levels and up‐regulating GSH in animal models.^80^ Pan et al highlighted the effects of various MT doses, showing that a range of MT concentrations (2.5, 5, 10 mg/kg) were effective in reducing hepatic steatosis and inflammation in HFD rats via increasing SOD and GSH activities, while only the high dose (10 mg/kg) of MT reduced MDA levels in liver tissue from NAFLD rats.^73^

**Table 3:** The potential mechanisms through which MT may attenuate NASH‐ and NAFLD‐induced liver injury

Animal	Treatment	HFD time	MT dose	MT time	MT route	Effect	Mechanism	Ref.
Mouse	HFD	15 wk	10 and 20 mg/kg/d	28 d	Intraperitoneal	Reduces hepatic fat deposition and inflammation; abolishes collagen deposition; prevents fibrosis progression	Enzymatic activity associated with the respiratory chain and TCA cycle↑; interaction between steatotic hepatocyte and HSCs↓	74
Rat	HFD	12 wk	2.5 or 5 or 10 mg/kg/d	12 wk	Intraperitoneal	Reduces hepatic steatosis and inflammation	SOD↑; GSH↑; MDA↓	76
Rat	HFD	4 or 8 or 12 wk	5 or 10 mg/kg/d	4 or 8 wk	Intraperitoneal	Decreases liver weight, liver weight/bodyweight ratio, portal vein pressure; reduces the expression of HFD‐induced plasma protein related to liver steatosis and the necrosis rate of liver cells; mitigates liver damage	MDA↓; GSH↑	77
Rat	HFD	10 wk	10 mg/kg/d	6 wk	Intraperitoneal	Reduces the HFD‐induced expression of plasma proteins related to liver steatosis and the necrosis rate of liver cells; mitigates liver damage	GPx3, serotransferrin, FBG β chain and C‐reactive protein ↑; complement factor B and APOE↑	78
Mouse	HFD	36 wk	10 mg/kg/d	12 wk	Per os	Increases insulin sensitivity; maintains glucose metabolism; reduces ALT, low‐density cholesterol, bodyweight and liver weight	Inflammatory factors (TNF‐α, IL‐1β and IL‐6)↓; phosphorylation of P38 and JNK1/2↓	80
Mouse	HFD	11 wk	50 or 100 mg/kg/d	10 wk	Per os	Ameliorates insulin resistance and lipid deposition	ER stress↓; AKT phosphorylation and fetuin‐A expression↑	81
Mouse	HFD	9 wk	100 mg/kg/d	8 wk	Per os	Reverses the pathological progress of NAFLD; improves hepatic morphological, ultrastructural and metabolic damage	Glycaemia↓; ER stress↓; mitochondria function↑; metabolic dysfunction↓; longevity of hepatocytes↑	82
Rat	HFD	16 wk	10 mg/kg/d	8 wk	Per os	Decreases the elevated activity of AST and ALT; attenuates the elevation of serum triglycerides levels; reduces the elevated HOMA‐IR index; decreases MDA; increases GSH; decreases steatosis and portal fibrosis	Serum total cholesterol and triglycerides↓	83
Rat	MCDD	4 wk	50 mg/kg/d	4 wk	Intraperitoneal	Decreases the number of TUNEL‐positive cells as well as hepatocyte apoptosis and NASH grade	MDA↑; GSH and SOD↑, inflammatory factors (IL‐1β, IL‐6 and TNF‐α)↓	15
Rat	Dexamethasone injection	Whole pregnancy period	1 mg/kg/d	Pregnancy period	Per os	Attenuates liver steatosis	Caspase 3↓; TNF‐α↓; reverses the methylation of leptin	84
Rat	Zucker diabetic fatty	N/A	10 mg/kg/d	6 wk	Per os	Alleviates liver steatosis and vacuolation; mitigates diabetes‐induced mitochondrial abnormalities as well as glycogen and lipid accumulation	ATP generation↑	85

Abbreviations: ALT, alanine transaminase; AST, aspartate aminotransferase; ER, endoplasmic reticulum; GSH, glutathione; HFD, high‐fat diet; HSC, hepatic stellate cell; IL, interleukin; MDA, malondialdehyde; MCDD, methionine‐ and choline‐deficient diet; MT, melatonin; NAFLD, nonalcoholic fatty liver disease; NASH, nonalcoholic steatohepatitis; SOD, superoxide dismutase; TNF, tumour necrosis factor.

Melatonin was also found to maintain liver function with decreased levels of ALT, AST and alkaline phosphatase; increase antioxidative functions including down‐regulation of MDA and up‐regulation of GSH and SOD; decrease inflammatory factors including IL‐1β, IL‐6 and TNF‐α; and consequently decrease the number of TUNEL‐positive cells as well as hepatocyte apoptosis and NASH grades in rats fed a methionine‐ and choline‐deficient diet.^12^ Prenatal glucocorticoids induced liver steatosis and apoptosis in neonatal rats, while MT reversed the methylation of leptin and decreased levels of caspase 3 and TNF‐α to attenuate liver steatosis in these neonatal rats.^81^ Furthermore, MT treatment alleviates liver steatosis and vacuolation and mitigates diabetes‐induced mitochondrial abnormalities as well as glycogen and lipid accumulation by improving ATP generation in Zucker diabetic fatty rats.^82^ To this end, MT significantly maintained the homeostasis of the fatty liver not only by regulating oxidative stress but also by decreasing the levels of proinflammatory cytokines. Although MT reduced the mean liver weights and ratios of liver to bodyweight and decreased hepatic steatosis in rats fed an HFD, there was no evidence showing that MT reversed established steatosis.^83^

### 5.4 Alcohol‐induced liver fibrosis

Alcohol abuse leads to asymptomatic and reversible alcoholic liver disease, and sustained alcohol consumption also directly results in irreversible liver fibrosis. After ingestion, alcohol is metabolized into the intermediate product acetaldehyde, which participates in the development of hepatic fibrogenesis via activation of the TGF‐β/SMAD signalling pathway,^84^ which then activates HSCs by inhibiting autophagy and ER stress. In addition, cytochrome P450 2E1 completes a crucial step in alcohol‐induced fibrogenesis by promoting overexpression of ROS in the liver,^85^ ultimately leading to ECM deposition and alcoholic liver cirrhosis.^86^ Ethanol can significantly up‐regulate NF‐κB translocation and MMP‐9 expression but down‐regulate TIMP‐1 expression, while MT prevents all these changes in addition to alcohol‐induced liver injury in mice.^87^ Given that alcohol‐induced liver cirrhosis is highly dependent on an ROS‐related mechanism, MT may serve as a potential agent to inhibit the alcohol‐induced pathogenetic process, including elevation of aminotransferases, triglyceride and hepatic steatosis, by up‐regulating phosphorylation of AMPK and SOD in alcohol‐treated rats.^88^ Hu et al demonstrated that MT maintained liver function and reduced the severity of hepatic cell damage and steatosis by reducing inflammatory cell infiltration, tissue lipid peroxidation, neutrophil infiltration and hepatocyte apoptosis. Moreover, MT treatment inhibits the release of ROS and TNF‐α in KCs isolated from ethanol‐fed mice.^89^

## 6 COMBINING MT AND MESENCHYMAL STEM CELLS TO REVERSE LIVER CIRRHOSIS

It is well known that mesenchymal stem cells (MSCs) can improve liver function and eliminate liver injury in human and animal models with hepatic fibrosis, but harsh microenvironments in vitro and in vivo reduce the therapeutic effects of MSCs. Current evidence suggests that MT may improve MSC stemness or MSC transplantation efficacy by enhancing antioxidant capacity and anti‐inflammation capacity. MT promoted the hepatic differentiation of MSCs and significantly increased bone morphogenetic protein‐2 expression and SMAD1/5/8 phosphorylation by activating the p38, extracellular signal‐regulated kinase (ERK) and NF‐κB signalling pathways. Moreover, the combined transplantation of MT and MSCs suppressed liver fibrosis in mice and restored liver function significantly more than MSC transplantation alone or MT treatment alone.^90^ Preconditioning with MT significantly improved the homing ability of MSCs in vivo, up‐regulated the expression of MMPs and Bcl2, and decreased the expression of TGF‐β1 and Bax, consequently restoring glycogen storage and decreasing collagen and lipid deposition in the fibrotic liver.^91^ Mortezaee et al asserted that preconditioning with 5 µmol/L MT for several passages did not alter MSC features in vitro and exerted no additional effects on reversing the pathological progress of CCl_4_‐induced liver fibrosis, although MT pretreatment significantly increased the homing ability of MSCs after transplantation in vivo.^92^ Both MT and MSCs can protect liver tissue from injury by enhancing antioxidant, anti‐inflammatory and antiapoptotic processes; thus, the combination of MT and MSCs is more effective than either agent alone in hindering the progression of liver cirrhosis.

## 7 CONCLUSIONS

Melatonin exerts protective effects by inhibiting oxidative stress, inflammatory signalling, autophagy flux, hepatocyte apoptosis and epithelial cell injury; thus, it attenuates the activation of HSCs and the proliferation of fibrogenic effector cells, ultimately reducing ECM deposition. Potentially, re‐establishment of the light/dark cycle and the circadian rhythm may increase the endogenous MT level and increase the therapeutic effects of MT in reversing the progression of fibrosis. The combination of MSC transplantation and MT administration will have stronger therapeutic effects than either strategy alone in inhibiting the initiation of HSC activation and ECM deposition. However, the majority of studies concerning MT therapy are conducted in animal models rather than human beings, leaving the therapeutic effects of MT on human liver fibrosis currently undefined. As we have discussed, MT is an effective antioxidative and anti‐inflammatory agent for eliminating cirrhotic liver injury and has great potential for application in the pharmaceutical industry.

## CONFLICT OF INTEREST

The authors declare no conflict of interest.

## AUTHOR CONTRIBUTIONS

Chenxia Hu wrote the manuscript; Lingfei Zhao revised the manuscript; Jingjing Tao collected the data; Lanjuan Li contributed to manuscript conception; and Chenxia Hu and Lanjuan Li provided financial support for the study. All authors have read and approved the final manuscript.

## References

[1] Yang X , He C , Zhu L , et al. Comparative analysis of regulatory role of Notch signaling pathway in 8 types liver cell during liver regeneration. Biochem Genet. 2019;57:1‐19. 29961162 10.1007/s10528-018-9869-2

[2] Zhou WC , Zhang QB , Qiao L . Pathogenesis of liver cirrhosis. World J Gastroenterol. 2014;20:7312‐7324. 24966602 10.3748/wjg.v20.i23.7312 PMC4064077

[3] Mafanda EK , Kandhi R , Bobbala D , et al. Essential role of suppressor of cytokine signaling 1 (SOCS1) in hepatocytes and macrophages in the regulation of liver fibrosis. Cytokine. 2018 pii: S1043-4666(18)30330-2. 10.1016/j.cyto.2018.07.032 30097285

[4] Wehr A , Baeck C , Heymann F , et al. Chemokine receptor CXCR4‐dependent hepatic NK T cell accumulation promotes inflammation and liver fibrosis. J Immunol. 2013;190:5226‐5236. 23596313 10.4049/jimmunol.1202909

[5] Forman HJ . Redox signaling: an evolution from free radicals to aging. Free Radic Biol Med. 2016;97:398‐407. 27393004 10.1016/j.freeradbiomed.2016.07.003 PMC4996735

[6] Reiter RJ . Pineal melatonin: cell biology of its synthesis and of its physiological interactions. Endocr Rev. 1991;12:151‐180. 1649044 10.1210/edrv-12-2-151

[7] Acuña‐Castroviejo D , Escames G , Venegas C , et al. Extrapineal melatonin: sources, regulation, and potential functions. Cell Mol Life Sci. 2014;71:2997‐3025. 24554058 10.1007/s00018-014-1579-2 PMC11113552

[8] Kennaway DJ . Are the proposed benefits of melatonin‐rich foods too hard to swallow? Crit Rev Food Sci Nutr. 2017;57:958‐962. 25975843 10.1080/10408398.2014.962686

[9] Ren W , Liu G , Chen S , et al. Melatonin signaling in T cells: functions and applications. J Pineal Res. 2017;62:e12394. 10.1111/jpi.12394 28152213

[10] Claustrat B , Brun J , Chazot G . The basic physiology and pathophysiology of melatonin. Sleep Med Rev. 2005;9:11‐24. 15649735 10.1016/j.smrv.2004.08.001

[11] Reiter RJ , Mayo JC , Tan D‐X , Sainz RM , Alatorre‐Jimenez M , Qin L . Melatonin as an antioxidant: under promises but over delivers. J Pineal Res. 2016;61:253‐278. 27500468 10.1111/jpi.12360

[12] Tahan V , Atug O , Akin H , et al. Melatonin ameliorates methionine‐ and choline‐deficient diet‐induced nonalcoholic steatohepatitis in rats. J Pineal Res. 2009;46:401‐407. 19552763 10.1111/j.1600-079X.2009.00676.x

[13] Wang H , Wei W , Wang N‐P , et al. Melatonin ameliorates carbon tetrachloride‐induced hepatic fibrogenesis in rats via inhibition of oxidative stress. Life Sci. 2005;77:1902‐1915. 15925388 10.1016/j.lfs.2005.04.013

[14] Aktas C , Kanter M , Erboga M , Mete R , Oran M . Melatonin attenuates oxidative stress, liver damage and hepatocyte apoptosis after bile‐duct ligation in rats. Toxicol Ind Health. 2014;30:835‐844. 23095487 10.1177/0748233712464811

[15] Hong RT , Xu JM , Mei Q . Melatonin ameliorates experimental hepatic fibrosis induced by carbon tetrachloride in rats. World J Gastroenterol. 2009;15:1452‐1458. 19322917 10.3748/wjg.15.1452 PMC2669124

[16] Czaja AJ . Hepatic inflammation and progressive liver fibrosis in chronic liver disease. World J Gastroenterol. 2014;20:2515‐2532. 24627588 10.3748/wjg.v20.i10.2515 PMC3949261

[17] Guicciardi ME , Gores GJ . Apoptosis as a mechanism for liver disease progression. Semin Liver Dis. 2010;30:402‐410. 20960379 10.1055/s-0030-1267540 PMC3071245

[18] Xie G , Wang X , Wang L , et al. Role of differentiation of liver sinusoidal endothelial cells in progression and regression of hepatic fibrosis in rats. Gastroenterology. 2012;142:918‐927.e916. 22178212 10.1053/j.gastro.2011.12.017 PMC3618963

[19] Wallace MC , Friedman SL , Mann DA . Emerging and disease‐specific mechanisms of hepatic stellate cell activation. Semin Liver Dis. 2015;35:107‐118. 25974897 10.1055/s-0035-1550060

[20] Berardis S , Dwisthi Sattwika P , Najimi M , Sokal EM . Use of mesenchymal stem cells to treat liver fibrosis: current situation and future prospects. World J Gastroenterol. 2015;21:742‐758. 25624709 10.3748/wjg.v21.i3.742 PMC4299328

[21] Boker KH , Pehle B , Steinmetz C , et al. Tissue inhibitors of metalloproteinases in liver and serum/plasma in chronic active hepatitis C and HCV‐induced cirrhosis. Hepatogastroenterology. 2000;47:812‐819. 10919037

[22] Kelly JD , Haldeman BA , Grant FJ , et al. Platelet‐derived growth factor (PDGF) stimulates PDGF receptor subunit dimerization and intersubunit trans‐phosphorylation. J Biol Chem. 1991;266:8987‐8992. 1709159

[23] Forbes SJ , Russo FP , Rey V , et al. A significant proportion of myofibroblasts are of bone marrow origin in human liver fibrosis. Gastroenterology. 2004;126:955‐963. 15057733 10.1053/j.gastro.2004.02.025

[24] Wells RG , Kruglov E , Dranoff JA . Autocrine release of TGF‐beta by portal fibroblasts regulates cell growth. FEBS Lett. 2004;559:107‐110. 14960316 10.1016/S0014-5793(04)00037-7

[25] Paik Y‐H , Kim J , Aoyama T , De Minicis S , Bataller R , Brenner DA . Role of NADPH oxidases in liver fibrosis. Antioxid Redox Signal. 2014;20:2854‐2872. 24040957 10.1089/ars.2013.5619 PMC4026397

[26] Lee YA , Friedman SL . Reversal, maintenance or progression: what happens to the liver after a virologic cure of hepatitis C? Antiviral Res. 2014;107:23‐30. 24726738 10.1016/j.antiviral.2014.03.012 PMC4050744

[27] Wang P , Koyama Y , Liu X , et al. Promising therapy candidates for liver fibrosis. Front Physiol. 2016;7:47. 26909046 10.3389/fphys.2016.00047 PMC4754444

[28] Steindl PE , Finn B , Bendok B , et al. Disruption of the diurnal rhythm of plasma melatonin in cirrhosis. Ann Intern Med. 1995;123:274‐277. 7611593 10.7326/0003-4819-123-4-199508150-00005

[29] Iguchi H , Kato KI , Ibayashi H . Melatonin serum levels and metabolic clearance rate in patients with liver cirrhosis. J Clin Endocrinol Metab. 1982;54:1025‐1027. 7061695 10.1210/jcem-54-5-1025

[30] De Rui M , Middleton B , Sticca A , et al. Sleep and circadian rhythms in hospitalized patients with decompensated cirrhosis: effect of light therapy. Neurochem Res. 2015;40:284‐292. 25135598 10.1007/s11064-014-1414-z

[31] Montagnese S , Middleton B , Mani AR , Skene DJ , Morgan MY . On the origin and the consequences of circadian abnormalities in patients with cirrhosis. Am J Gastroenterol. 2010;105:1773‐1781. 20332771 10.1038/ajg.2010.86

[32] Amodio P . Current diagnosis and classification of hepatic encephalopathy. J Clin Exp Hepatol. 2018;8:432‐437. 30564001 10.1016/j.jceh.2018.07.001 PMC6286442

[33] Sherlock S , Summerskill W , White LP , Phear EA . Portal‐systemic encephalopathy; neurological complications of liver disease. Lancet. 1954;267:454‐457. 13193045

[34] Velissaris D , Karanikolas M , Kalogeropoulos A , et al. Pituitary hormone circadian rhythm alterations in cirrhosis patients with subclinical hepatic encephalopathy. World J Gastroenterol. 2008;14:4190‐4195. 18636665 10.3748/wjg.14.4190 PMC2725381

[35] Chojnacki C , Romanowski M , Winczyk K , Błasiak J , Chojnacki J . Melatonin levels in serum and ascitic fluid of patients with hepatic encephalopathy. Gastroenterol Res Pract. 2012;2012:510764. 23346104 10.1155/2012/510764 PMC3546494

[36] Chojnacki C , Walecka‑Kapica E , Klupińska G , et al. Serotonin and melatonin secretion and metabolism in patients with liver cirrhosis. Pol Arch Med Wewn. 2012;122:392‐397. 22814406

[37] Chojnacki C , Wachowska‐Kelly P , Błasiak J , Reiter RJ , Chojnacki J . Melatonin secretion and metabolism in patients with hepatic encephalopathy. J Gastroenterol Hepatol. 2013;28:342‐347. 23190028 10.1111/jgh.12055

[38] Celinski K , Konturek PC , Slomka M , et al. Altered basal and postprandial plasma melatonin, gastrin, ghrelin, leptin and insulin in patients with liver cirrhosis and portal hypertension without and with oral administration of melatonin or tryptophan. J Pineal Res. 2009;46:408‐414. 19552764 10.1111/j.1600-079X.2009.00677.x

[39] Cichoz‐Lach H , Celinski K , Konturek PC , et al. The effects of L‐tryptophan and melatonin on selected biochemical parameters in patients with steatohepatitis. J Physiol Pharmacol. 2010;61:577‐580. 21081801

[40] Celinski K , Konturek PC , Slomka M , et al. Effects of treatment with melatonin and tryptophan on liver enzymes, parameters of fat metabolism and plasma levels of cytokines in patients with non‐alcoholic fatty liver disease–14 months follow up. J Physiol Pharmacol. 2014;65:75‐82. 24622832

[41] Gonciarz M , Gonciarz Z , Bielanski W , et al. The effects of long‐term melatonin treatment on plasma liver enzymes levels and plasma concentrations of lipids and melatonin in patients with nonalcoholic steatohepatitis: a pilot study. J Physiol Pharmacol. 2012;63:35‐40. 22460459

[42] Pakravan H , Ahmadian M , Fani A , Aghaee D , Brumanad S , Pakzad B . The effects of melatonin in patients with nonalcoholic fatty liver disease: a randomized controlled trial. Adv Biomed Res. 2017;6:40. 28503495 10.4103/2277-9175.204593 PMC5414412

[43] Ogeturk M , Kus I , Pekmez H , Yekeler H , Sahin S , Sarsilmaz M . Inhibition of carbon tetrachloride‐mediated apoptosis and oxidative stress by melatonin in experimental liver fibrosis. Toxicol Ind Health. 2008;24:201‐208. 19022872 10.1177/0748233708093725

[44] González‐Fernández B , Sánchez DI , Crespo I , et al. Melatonin attenuates dysregulation of the circadian clock pathway in mice with CCl_4_‐induced fibrosis and human hepatic stellate cells. Front Pharmacol. 2018;9:556. 29892224 10.3389/fphar.2018.00556 PMC5985434

[45] Crespo I , San‐Miguel B , Fernández A , Ortiz de Urbina J , González‐Gallego J , Tuñón MJ . Melatonin limits the expression of profibrogenic genes and ameliorates the progression of hepatic fibrosis in mice. Transl Res. 2015;165:346‐357. 25445210 10.1016/j.trsl.2014.10.003

[46] González‐Fernández B , Sánchez DI , Crespo I , et al. Inhibition of the SphK1/S1P signaling pathway by melatonin in mice with liver fibrosis and human hepatic stellate cells. BioFactors. 2017;43:272‐282. 27801960 10.1002/biof.1342

[47] Bona S , Rodrigues G , Moreira AJ , et al. Antifibrogenic effect of melatonin in rats with experimental liver cirrhosis induced by carbon tetrachloride. JGH Open. 2018;2:117‐123. 30483575 10.1002/jgh3.12055 PMC6206983

[48] Choi HS , Kang JW , Lee SM . Melatonin attenuates carbon tetrachloride‐induced liver fibrosis via inhibition of necroptosis. Transl Res. 2015;166:292‐303. 25936762 10.1016/j.trsl.2015.04.002

[49] San‐Miguel B , Crespo I , Sánchez DI , et al. Melatonin inhibits autophagy and endoplasmic reticulum stress in mice with carbon tetrachloride‐induced fibrosis. J Pineal Res. 2015;59:151‐162. 25958928 10.1111/jpi.12247

[50] Kang JW , Hong JM , Lee SM . Melatonin enhances mitophagy and mitochondrial biogenesis in rats with carbon tetrachloride‐induced liver fibrosis. J Pineal Res. 2016;60:383‐393. 26882442 10.1111/jpi.12319

[51] Mortezaee K , Majidpoor J , Daneshi E , et al. Post‐treatment of melatonin with CCl_4_ better reduces fibrogenic and oxidative changes in liver than melatonin co‐treatment. J Cell Biochem. 2018;119:1716‐1725. 28782839 10.1002/jcb.26331

[52] Lebda MA , Sadek KM , Abouzed TK , Tohamy HG , El‐Sayed YS . Melatonin mitigates thioacetamide‐induced hepatic fibrosis via antioxidant activity and modulation of proinflammatory cytokines and fibrogenic genes. Life Sci. 2018;192:136‐143. 29180002 10.1016/j.lfs.2017.11.036

[53] Czechowska G , Celinski K , Korolczuk A , et al. Protective effects of melatonin against thioacetamide‐induced liver fibrosis in rats. J Physiol Pharmacol. 2015;66:567‐579. 26348081

[54] Cruz A , Padillo FJ , Torres E , et al. Melatonin prevents experimental liver cirrhosis induced by thioacetamide in rats. J Pineal Res. 2005;39:143‐150. 16098091 10.1111/j.1600-079X.2005.00227.x

[55] Tahan V , Ozaras R , Canbakan B , et al. Melatonin reduces dimethylnitrosamine‐induced liver fibrosis in rats. J Pineal Res. 2004;37:78‐84. 15298665 10.1111/j.1600-079X.2004.00137.x

[56] Pinzani M , Luong TV . Pathogenesis of biliary fibrosis. Biochim Biophys Acta. 2018;1864:1279‐1283. 10.1016/j.bbadis.2017.07.026 28754450

[57] Mills PR , Skerrow CJ , MacKie RM . Melanin pigmentation of the skin in primary biliary cirrhosis. J Cutan Pathol. 1981;8:404‐410. 6278001 10.1111/j.1600-0560.1981.tb01029.x

[58] Lens S , Leoz M , Nazal L , Bruguera M , Parés A . Bezafibrate normalizes alkaline phosphatase in primary biliary cirrhosis patients with incomplete response to ursodeoxycholic acid. Liver Int. 2014;34:197‐203. 23998489 10.1111/liv.12290

[59] Gylling H , Farkkila M , Vuoristo M , et al. Metabolism of cholesterol and low‐ and high‐density lipoproteins in primary biliary cirrhosis: cholesterol absorption and synthesis related to lipoprotein levels and their kinetics. Hepatology. 1995;21:89‐95. 7806174

[60] Li Y , Wang W , Tang L , et al. Chemokine (C‐X‐C motif) ligand 13 promotes intrahepatic chemokine (C‐X‐C motif) receptor 5+ lymphocyte homing and aberrant B‐cell immune responses in primary biliary cirrhosis. Hepatology. 2015;61:1998‐2007. 25627620 10.1002/hep.27725 PMC4441570

[61] Wu N , Meng F , Zhou T , et al. Prolonged darkness reduces liver fibrosis in a mouse model of primary sclerosing cholangitis by miR‐200b down‐regulation. FASEB J. 2017;31:4305‐4324. 28634212 10.1096/fj.201700097R PMC5987749

[62] Sezer A , Hatipoglu AR , Usta U , Altun G , Sut N . Effects of intraperitoneal melatonin on caustic sclerosing cholangitis due to scolicidal solution in a rat model. Curr Ther Res Clin Exp. 2010;71:118‐128. 24683258 10.1016/j.curtheres.2010.03.004 PMC3967282

[63] Veidal SS , Vassiliadis E , Bay‐Jensen A‐C , Tougas G , Vainer B , Karsdal MA . Procollagen type I N‐terminal propeptide (PINP) is a marker for fibrogenesis in bile duct ligation‐induced fibrosis in rats. Fibrogenesis Tissue Repair. 2010;3:5. 20359335 10.1186/1755-1536-3-5 PMC2860343

[64] Renzi A , Glaser S , DeMorrow S , et al. Melatonin inhibits cholangiocyte hyperplasia in cholestatic rats by interaction with MT1 but not MT2 melatonin receptors. Am J Physiol Gastrointest Liver Physiol. 2011;301:G634‐G643. 21757639 10.1152/ajpgi.00206.2011 PMC3191552

[65] Colares JR , Schemitt EG , Hartmann RM , et al. Antioxidant and anti‐inflammatory action of melatonin in an experimental model of secondary biliary cirrhosis induced by bile duct ligation. World J Gastroenterol. 2016;22:8918‐8928. 27833383 10.3748/wjg.v22.i40.8918 PMC5083797

[66] McMillin M , DeMorrow S , Glaser S , et al. Melatonin inhibits hypothalamic gonadotropin‐releasing hormone release and reduces biliary hyperplasia and fibrosis in cholestatic rats. Am J Physiol Gastrointest Liver Physiol. 2017;313:G410‐G418. 28751425 10.1152/ajpgi.00421.2016 PMC5792219

[67] Tahan G , Akin H , Aydogan F , et al. Melatonin ameliorates liver fibrosis induced by bile‐duct ligation in rats. Can J Surg. 2010;53:313‐318. 20858375 PMC2947113

[68] Tain Y‐L , Hsieh C‐S , Chen C‐C , Sheen J‐M , Lee C‐T , Huang L‐T . Melatonin prevents increased asymmetric dimethylarginine in young rats with bile duct ligation. J Pineal Res. 2010;48:212‐221. 20210851 10.1111/j.1600-079X.2010.00745.x

[69] Hsu M‐H , Chen Y‐C , Sheen J‐M , Li S‐W , Huang L‐T . Melatonin prevented spatial deficits and increases in brain asymmetric dimethylarginine in young bile duct ligation rats. NeuroReport. 2018;29:541‐546. 29384993 10.1097/WNR.0000000000000972 PMC6023590

[70] Huang L‐T , Tiao M‐M , Tain Y‐L , Chen C‐C , Hsieh C‐S . Melatonin ameliorates bile duct ligation‐induced systemic oxidative stress and spatial memory deficits in developing rats. Pediatr Res. 2009;65:176‐180. 19047958 10.1203/PDR.0b013e31818d5bc7

[71] Das N , Mandala A , Naaz S , et al. Melatonin protects against lipid‐induced mitochondrial dysfunction in hepatocytes and inhibits stellate cell activation during hepatic fibrosis in mice. J Pineal Res. 2017;62:e12404. 10.1111/jpi.12404 28247434

[72] Zhou H , Du W , Li YE , et al. Effects of melatonin on fatty liver disease: the role of NR4A1/DNA‐PKcs/p53 pathway, mitochondrial fission, and mitophagy. J Pineal Res. 2018;64:e12450. 10.1111/jpi.12450 28981157

[73] Pan M , Song Y‐L , Xu J‐M , Gan H‐Z . Melatonin ameliorates nonalcoholic fatty liver induced by high‐fat diet in rats. J Pineal Res. 2006;41:79‐84. 16842545 10.1111/j.1600-079X.2006.00346.x

[74] Hatzis G , Ziakas P , Kavantzas N , et al. Melatonin attenuates high fat diet‐induced fatty liver disease in rats. World J Hepatol. 2013;5:160‐169. 23671720 10.4254/wjh.v5.i4.160 PMC3648647

[75] Wongchitrat P , Klosen P , Pannengpetch S , Kitidee K , Govitrapong P , Isarankura‐Na‐Ayudhya C . High‐fat diet‐induced plasma protein and liver changes in obese rats can be attenuated by melatonin supplementation. Nutr Res. 2017;42:51‐63. 28633871 10.1016/j.nutres.2017.04.011

[76] Shajari S , Laliena A , Heegsma J , et al. Melatonin suppresses activation of hepatic stellate cells through RORalpha‐mediated inhibition of 5‐lipoxygenase. J Pineal Res. 2015;59:391‐401. 26308880 10.1111/jpi.12271

[77] Sun H , Wang X , Chen J , et al. Melatonin improves non‐alcoholic fatty liver disease via MAPK‐JNK/P38 signaling in high‐fat‐diet‐induced obese mice. Lipids Health Dis. 2016;15:202. 27876064 10.1186/s12944-016-0370-9 PMC5120511

[78] Heo J‐I , Yoon DW , Yu JH , et al. Melatonin improves insulin resistance and hepatic steatosis through attenuation of alpha‐2‐HS‐glycoprotein. J Pineal Res. 2018;65:e12493. 29607540 10.1111/jpi.12493

[79] Stacchiotti A , Favero G , Lavazza A , et al. Hepatic macrosteatosis is partially converted to microsteatosis by melatonin supplementation in ob/ob mice non‐alcoholic fatty liver disease. PLoS ONE. 2016;11:e0148115. 26824477 10.1371/journal.pone.0148115 PMC4732686

[80] Zaitone S , Hassan N , El‐Orabi N , El‐Awady E‐S . Pentoxifylline and melatonin in combination with pioglitazone ameliorate experimental non‐alcoholic fatty liver disease. Eur J Pharmacol. 2011;662:70‐77. 21549113 10.1016/j.ejphar.2011.04.049

[81] Tiao M‐M , Huang L‐T , Chen C‐J , et al. Melatonin in the regulation of liver steatosis following prenatal glucocorticoid exposure. Biomed Res Int. 2014;2014:942172. 24822223 10.1155/2014/942172 PMC4005100

[82] Agil A , El‐Hammadi M , Jiménez‐Aranda A , et al. Melatonin reduces hepatic mitochondrial dysfunction in diabetic obese rats. J Pineal Res. 2015;59:70‐79. 25904243 10.1111/jpi.12241

[83] Sun H , Huang FF , Qu S . Melatonin: a potential intervention for hepatic steatosis. Lipids Health Dis. 2015;14:75. 26199093 10.1186/s12944-015-0081-7 PMC4511016

[84] Chen N , Geng Q , Zheng J , et al. Suppression of the TGF‐beta/Smad signaling pathway and inhibition of hepatic stellate cell proliferation play a role in the hepatoprotective effects of curcumin against alcohol‐induced hepatic fibrosis. Int J Mol Med. 2014;34:1110‐1116. 25069637 10.3892/ijmm.2014.1867

[85] Kim DK , Kim YH , Jang HH , et al. Estrogen‐related receptor gamma controls hepatic CB1 receptor‐mediated CYP2E1 expression and oxidative liver injury by alcohol. Gut. 2013;62:1044‐1054. 23023167 10.1136/gutjnl-2012-303347 PMC3812689

[86] Mir RA , Chauhan SS . Down regulation of a matrix degrading cysteine protease cathepsin L, by acetaldehyde: role of C/EBPalpha. PLoS ONE. 2011;6:e20768. 21687683 10.1371/journal.pone.0020768 PMC3110794

[87] Mishra A , Paul S , Swarnakar S . Downregulation of matrix metalloproteinase‐9 by melatonin during prevention of alcohol‐induced liver injury in mice. Biochimie. 2011;93:854‐866. 21354255 10.1016/j.biochi.2011.02.007

[88] Rui B‐B , Chen H , Jang L , et al. Melatonin upregulates the activity of AMPK and attenuates lipid accumulation in alcohol‐induced rats. Alcohol Alcohol. 2016;51:11‐19. 26564773 10.1093/alcalc/agv126

[89] Hu S , Yin S , Jiang X , Huang D , Shen G . Melatonin protects against alcoholic liver injury by attenuating oxidative stress, inflammatory response, and apoptosis. Eur J Pharmacol. 2009;616:287‐292. 19576882 10.1016/j.ejphar.2009.06.044

[90] Cho Y‐A , Noh K , Jue S‐S , Lee S‐Y , Kim E‐C . Melatonin promotes hepatic differentiation of human dental pulp stem cells: clinical implications for the prevention of liver fibrosis. J Pineal Res. 2015;58:127‐135. 25431168 10.1111/jpi.12198

[91] Mortezaee K , Khanlarkhani N , Sabbaghziarani F , et al. Preconditioning with melatonin improves therapeutic outcomes of bone marrow‐derived mesenchymal stem cells in targeting liver fibrosis induced by CCl_4_ . Cell Tissue Res. 2017;369:303‐312. 28413861 10.1007/s00441-017-2604-1

[92] Mortezaee K , Pasbakhsh P , Ragerdi Kashani I , et al. Melatonin pretreatment enhances the homing of bone marrow‐derived mesenchymal stem cells following transplantation in a rat model of liver fibrosis. Iran Biomed J. 2016;20:207‐216. 27130910 10.7508/ibj.2016.04.004 PMC4983675

